# Nontraditional Antibiotics—Challenges and Triumphs

**DOI:** 10.3390/antibiotics9040169

**Published:** 2020-04-09

**Authors:** Karl A. Hansford

**Affiliations:** Centre for Superbug Solutions, Institute for Molecular Bioscience, University of Queensland, St Lucia 4072, Australia; k.hansford@uq.edu.au

The pursuit of nontraditional antibiotics is becoming an increasingly important means to tackle seemingly insurmountable challenges faced by contemporary antibiotic researchers as they overcome the shifting landscape of bacterial pathogenesis, particularly for Gram-negative bacteria. Defined as therapeutic modalities that do not fit within the traditional direct-acting small molecule paradigm, the scientific merit, translational potential and likely pitfalls of nontraditional approaches was recently reviewed [1]. Encouragingly, there is an unprecedented level of activity around nontraditional therapies based on a recent analysis of the global preclinical antibacterial pipeline [2]. Although the future potential of nontraditional therapies is viewed with cautious optimism, adequate resourcing will be critical to demonstrate clinical impact, especially when many of the translational criteria associated with direct-acting small molecule therapeutics will likely be incongruent with the nontraditional development pathway. This is reinforced by the sobering point that nontraditional therapies will likely be used as adjunctive therapies alongside antibiotics, rather than replacing them, clouding clinical endpoints for regulatory approval [3]. Thus, considerable work is still required to navigate the tortuous pathway to the regulatory approval and future acceptance of nontraditional therapies in clinical practice. 

This special issue of *Antibiotics* is dedicated to providing a compendium of the latest developments in the field from a diverse cross section of researchers working at the forefront of antibiotic discovery. With a contribution of seven articles (one communication, three reviews, one commentary, one concept paper and one perspective), the authors are sincerely thanked for their efforts.

The concept paper by Azeredo da Silveira and Shorr provides an excellent case study on CAL02, a liposome-derived anti-virulence trap designed to sequester bacterial toxins by acting as a decoy mimic of cellular lipid platforms [4]. The three review articles by Zurawski and McLendon [5], Nikolich and Filippov [6], and Ghose and Euler [7] give a superb timely survey of how monoclonal antibodies, bacteriophages and lysins, respectively, are currently being explored in the treatment of bacterial infections. The utility and future potential of antibodies to treat bacterial infections is only just being realized now, despite a successful history in other therapeutic areas. Similarly, despite widespread use in Eastern European countries for decades, bacteriophage therapy is only just being explored in the West. Zurawski and McLendon [5] and Nikolich and Filippov [6] provide a contemporary account through the drug development lens, highlighting previous successes and failures, lessons learned and what we might expect in the coming years. Bacteriophage endolysins are another product that have been studied intensively, albeit generally against Gram-positive organisms due to the Gram-negative impermeability barrier. Speaking from a commercial drug developer’s viewpoint, Ghose and Euler give a unique detailed account of newly discovered lysins with Gram-negative activity currently in the early stages of development [7]. This will no doubt be of great interest to developers working in the field. Turning to small molecule therapeutics, Frei’s perspective on the untapped potential of metal complexes as antibiotics provides a compelling view that chemical classes often misconstrued as only being useful for certain applications should not be overlooked as potential sources of new chemotypes to fight bacterial infections [8]. Complementing this account is the commentary by Quave and Marquez, who highlight that it would be remiss to overlook the virtues of medical ethnobotany—the study of how people use plants in medicine—in the quest for new natural products to treat infections, with a particular emphasis on antifungals [9]. Finally, a communication by Gajdács and Spengler details the role of drug repurposing as a source of new virulence inhibitors [10]. By testing the utility of a diverse set of pharmacological agents as quorum sensing (QS)-inhibitors, they identified fourteen agents possessing dose-dependent QS-inhibitory activity in vitro, concluding that the QS-based modulation of bacterial virulence is a promising strategy warranting further investigation.

As this issue comes to a close, we face an unprecedented global challenge due to the coronavirus COVID-19 pandemic. The world has largely been ill prepared to manage this kind of onslaught, highlighting the catastrophic dangers of unforeseen biological threats. Naturally, antibiotics are being used to treat secondary infections in COVID-19 patients, leading to increased pressures on resistance selection, and the potential for critical shortages if supply chains are disrupted. Unlike COVID-19, drug-resistant infections have been on our radar for years. New, effective interventions are needed now, before we reach a tipping point. The articles in this special issue are a snapshot of the incredible work being undertaken by many talented and dedicated developers who choose to remain in the field for the long haul. It should serve to inspire us all. 
